# Preoperative X-ray C_2_C_6_AR is applicable for prediction of difficult laryngoscopy in patients with cervical spondylosis

**DOI:** 10.1186/s12871-021-01335-4

**Published:** 2021-04-12

**Authors:** Yang Zhou, Yongzheng Han, Zhengqian Li, Yuqing Zhao, Ning Yang, Taotao Liu, Min Li, Jun Wang, Xiangyang Guo, Mao Xu

**Affiliations:** 1 Department of Anesthesiology, Peking University Third Hospital, Peking University Health Science Center, 49 North Garden Road, Haidian District, Beijing, P.R. China; 2 Department of Radiology, Peking University Third Hospital, Peking University Health Science Center, Beijing, China

**Keywords:** X-ray, Difficult laryngoscopy, Cervical spondylosis, Radiologic indicators, Cervical mobility

## Abstract

**Background:**

Airway management is one of the most important techniques in anesthesia practice and inappropriate airway management is related with airway injury, brain hypoxia, and even death. The patients with cervical spondylosis are often confronted with difficult laryngoscopy who are more prone to appear difficult airway, so it is important to figure out valuable predictors of difficult laryngoscopy in these patients.

**Methods:**

We randomly enrolled 270 patients undergoing elective cervical spine surgery and analyzed the cervical mobility data in predicting difficult laryngoscopy. The preoperative X-ray radiological indicators were measured by an attending radiologist. Cormack-Lehane scales were assessed during intubation, and patients with a class III or IV view were assigned to the difficult laryngoscopy group.

**Results:**

Univariate analysis showed that the hyomental distance (HMD, the distance between the hyoid bone and the tip of the chin) and the hyomental distance ratio (HMDR, the ratio between HMD in the extension position and the one in the neutral position) might not be suitable indicators in patients with cervical spondylosis. Binary multivariate logistic regression (backward-Wald) analyses identified two independent correlative factors from the cervical mobility indicators that correlated best as a predictor of difficult laryngoscopy: modified Mallampati test (MMT) and C_2_C_6_AR (the ratio of the angle between a line passing through the bottom of the second cervical vertebra and a line passing through the bottom of the sixth cervical vertebra in the extension position and the one in the neutral position). The odds ratio (OR) and 95 % CI were 2.292(1.093–4.803) and 0.493 (0.306–0.793), respectively. C_2_C_6_AR exhibited the largest area under the curve (0.714; 95 % CI 0.633–0.794).

**Conclusions:**

C_2_C_6_AR based on preoperative X-ray images may be the most accurate predictor of cervical mobility indicators for difficult laryngoscopy in patients with cervical spondylosis.

**Trial registration:**

The study was registered at the Chinese Clinical Trial Registry (http://www.chictr.org.cn; identifier: ChiCTR-ROC-16,008,598) on June 6, 2016.

## Background

Airway management is one of the most important techniques in clinical anesthesia practice. Inappropriate airway management may lead to airway injury, brain hypoxia and airway management failure is the primary cause of anesthesia-related deaths. According to the recent ASA Closed Claims, the number of claims related to difficult tracheal intubation is comparable with the previous phase, however, outcomes remained poor and differed with a higher proportion of death (73 % in 2000 to 2012 vs. 42 % in 1993 to 1999) [1]. At present, the incidence rate of cervical spondylosis is increasing year by year, and these patients are often confronted with difficult laryngoscopy [2], with the incidence of difficult laryngoscopy to be 17.1 % [3], far more than the incidence of 5.8 % in the general population [4]. Although cervical spondylosis could be an alert signal for a predictable difficult airway, the difficulty of tracheal intubation is variable in different types of cervical spondylosis. Some of the patients are more prone to appear difficult laryngoscopy during tracheal intubation, which could even develop into the emergency airway, such as can’t intubation and can’t ventilation situation. There is still a lack of effective and specific evaluation methods for these patients, and it is fundamentally important to figure out the most valuable predictor of difficult laryngoscopy in patients with cervical spondylosis.

Mallampati III and IV are the most popular conventional predictors for difficult ventilation [5, 6], but their prognostic value for difficult laryngoscopy was poor. After a meta-analysis involving 177 088 patients, Lundstrøm et al [7] found that the modified Mallampati test (MMT) is inadequate as a stand-alone test of a difficult laryngoscopy with the predictive sensitivity of 0.35. To screen out the potential difficult airway patients with cervical spondylosis and avoid the unanticipated difficult airway, we had better make full use of the radiological images as indicators preoperatively. Difficult Airway Society 2015 guideline had pointed out that radiological examination which could provide more precise information regarding anatomical structures proved to be a suitable method for predicting a difficult airway [8]. In this study, we recruited patients diagnosed with cervical spondylotic radiculopathy or myelopathy, aimed to explore a valuable radiologic indicator to predict difficult laryngoscopy compared to physical examinations in patients with cervical spondylosis.

## Methods

The protocol was approved by the Medical Ethics Committee of the authors’ hospital (IRB00006761-2015021), and the informed consent forms were obtained from the patients. Patients with cervical spondylotic radiculopathy or myelopathy who needed to undergo elective cervical spine surgery under general anesthesia with oral endotracheal intubation were included. Exclusion criteria included pregnancy, cervical spondilolystesis, cervical segmental instability, anatomical abnormality like oropharyngeal mass or micrognathia, medical history of failed or difficult intubation. This study was approved by the ethics committee of the hospital and registered at the Chinese Clinical Trial Registry (http://www.chictr.org.cn; identifier: ChiCTR-ROC-16,008,598) on June 6, 2016.

MMT was assessed preoperatively. The patients sit upright with the head in a neutral position. The oropharyngeal structures were observed when the mouth was maximally opened and the tongue protruded by the anesthesiologist who sits opposite at eye level with a pen torch. The airway was classified according to the structures seen: class I, soft palate, fauces, uvula, pillars; class II, soft palate, fauces, uvula; class III, soft palate, base of the uvula; class IV, soft palate not visible at all [9].

**Fig. 1:**
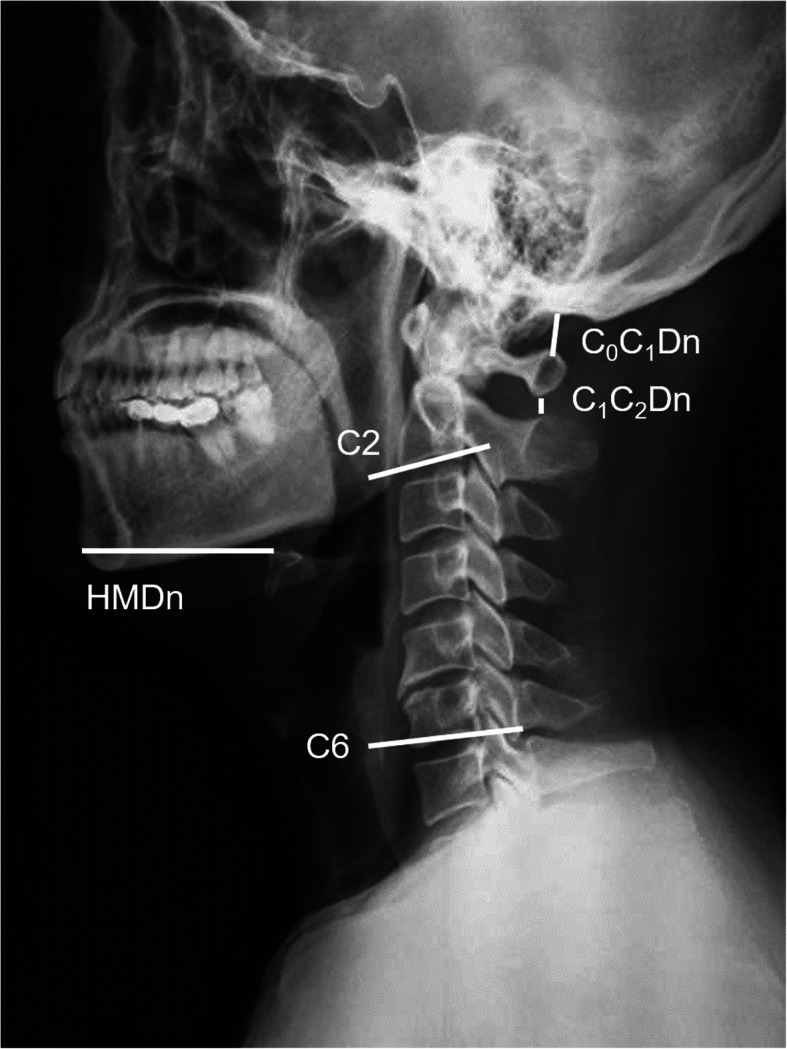
Lateral cervical X-ray film in the neutral positions. HMDn, the distance between the hyoid bone and the tip of the chin in the neutral position; C_0_C_1_Dn, the distance between the occipital bone and first cervical vertebra in the neutral position; C_1_C_2_Dn, the distance between the first cervical vertebra and the second cervical vertebra in the neutral position

**Fig. 2:**
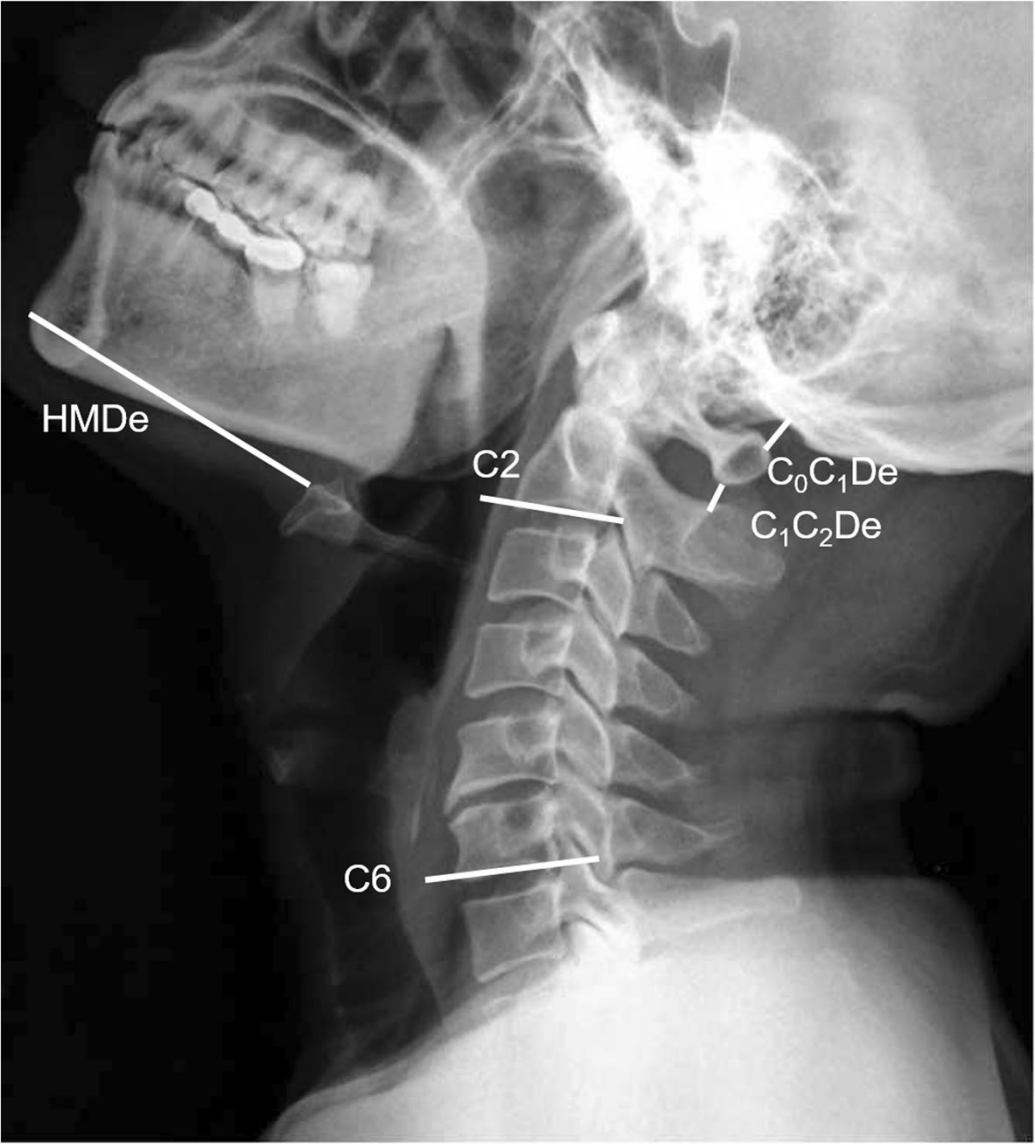
Lateral cervical X-ray film in the extension positions. HMDe, the distance between the hyoid bone and the tip of the chin in the extension position; C_0_C_1_De, the distance between the occipital bone and first cervical vertebra in the extension position; C_1_C_2_De, the distance between the first cervical vertebra and the second cervical vertebra in the extension position

The patients were routinely examined with cervical spine plain X-ray at lateral view both in neutral and extension positions. They were instructed to stand in a designated location and were requested to keep the upper cervical spines with retroversion as much as possible when extension applied and ordered not to move the lower cervical spines and shoulder muscles. All radiological indicators were measured using the radiography information system (Centricity RIS-IC CE V3.0; GE Healthcare, Little Chalfont, UK), by the same experienced radiologist who was blind to the anesthesia operation (Figs. 1 and 2).

All patients received standardised general anaesthesia with sufentanil (0.3 mcg/kg), propofol (2 mg/kg) and rocuronium (0.6 mg/kg). After muscle relaxation was achieved, the laryngoscopy view was assessed by a single experienced anesthesiologist who was blind to the preoperative radiological data using the Macintosh laryngoscope. The result was determined by the Cormack-Lehane (C-L) grade [10]. Patients with Cormack-Lehane grade 3 or 4 were assigned to the difficult laryngoscopy group, and patients with Cormack-Lehane grade 1 or 2 were assigned to the easy laryngoscopy group. Then, tracheal intubation was performed with a Macintosh laryngoscope or alternative device by the same anesthesiologist. In patients with a difficult airway, intubation was performed according to the Difficult Airway Society 2015 guidelines [8].

### Statistical analysis

The previous study had reported that the incidence of difficult laryngoscopy could be as high as 20 % [11]. A power calculation showed that 245 patients would be required to detect a difference in predictors between the difficult and easy laryngoscopy groups (α = 0.05 and β = 0.1). In consideration of a potential dropout, 270 patients were recruited for the study. Data were analyzed by SPSS software (Version 21.0; IBM Corp., USA). The data are expressed as the mean ± standard deviation (SD), the median and interquartile range (IQR), or the number (%). The Kolmogorov–Smirnov method was used to test the normality of all of the variables. Categorical variables were analyzed using a χ^2^ test, while continuous variables were analyzed using an independent-samples t-test. The Mann–Whitney U-test was used to analyze non-normal variables. Binary multivariate logistic regression analyses were performed. A receiver operating characteristic (ROC) curve and the area under the curve (AUC) was used to describe the discrimination abilities of the predictive indicators. Statistical significance was set at P < 0.05.

## Results

270 patients meeting the inclusion criteria were recruited from June 2016 to December 2016. The cervical mobility indicators assessed in this study are listed in Table 1. Three indicators were significantly different between the easy and difficult laryngoscopy groups: the MMT grade (*P* = 0.037), C_2_C_6_An (the angle between a line passing through the bottom of the second cervical vertebra and a line passing through the bottom of the sixth cervical vertebra in the neutral position, *P* = 0.013) and C_2_C_6_AR (the ratio of the angle between a line passing through the bottom of second cervical vertebra and a line passing through the bottom of sixth cervical vertebra in the extension position and the one in the neutral position, *P* < 0.001). There was a higher MMT grade (class III-IV) ratio, less C_2_C_6_An, and less C_2_C_6_AR in difficult laryngoscopy group patients versus the easy laryngoscopy group.

**Table 1:** Cervical mobility indicators to predict difficult laryngoscopy between the two groups of patients undergoing cervical spine surgery

Items	Easy laryngoscopy group (*n* = 230)	Difficult laryngoscopy group (*n* = 40)	Statistical Test	*P*-values
BMI	25.1 ± 3.3	25.7 ± 2.5	t=-1.127	0.261
**MMT (class I-II/class III-IV)**	**149(64.8)/81(35.2)**	**19(47.5)/21(52.5)**	**χ**^**2**^ **= 4.33**	**0.037**
HMDn (cm)	5.4 ± 0.8	5.3 ± 0.9	t = 0.777	0.438
HMDe (cm)	6.6 ± 1.0	6.5 ± 0.8	t = 1.073	0.285
HMDR	1.21 (0.19)	1.22 (0.13)	z=-0.382	0.703
C_0_C_1_Dn (mm)	6.7 ± 2.6	6.4 ± 3.1	t = 0.665	0.507
C_0_C_1_De (mm)	0.6 ± 0.3	0.5 ± 0.3	t = 1.249	0.213
C_0_C_1_DR	12.14 (10.29)	13.70 (10.10)	z = 0.490	0.624
C_1_C_2_Dn (mm)	4.6 (2.5)	5.0 (4.3)	z = 1.113	0.266
C_1_C_2_De (mm)	0.5 (0.3)	0.6 (0.3)	z = 1.087	0.277
C_1_C_2_DR	8.88 (5.36)	8.96 (6.51)	z = 0.259	0.796
**C**_**2**_**C**_**6**_**An (°)**	**9.0 (12.0)**	**13.5 (10.9)**	**z = 2.484**	**0.013**
C_2_C_6_Ae (°)	16.7 (13.6)	17.6 (16.1)	z=-0.572	0.568
**C**_**2**_**C**_**6**_**AR**	**1.80 (2.25)**	**1.26 (0.56)**	**z=-4.127**	**< 0.001**

*BMI* Body Mass Index; *MMT* modified Mallampati test; *HMDn/e* the distance between the hyoid bone and the tip of the chin in the neutral/ extension position; *HMDR* the ratio between HMDe and HMDn; *C*_*0*_*C*_*1*_*Dn/e* the distance between the occipital bone and the first cervical vertebra in the neutral/ extension position; *C*_*0*_*C*_*1*_*DR* the ratio between C_0_C_1_Dn and C_0_C_1_De; *C*_*1*_*C*_*2*_*Dn/e* the distance between first cervical vertebra and the second cervical vertebra in the neutral/ extension position; *C*_*1*_*C*_*2*_*DR* the ratio between C_1_C_2_Dn and C_1_C_2_De; *C*_*2*_*C*_*6*_*An/e* the angle between a line passing through the bottom of second cervical vertebra and a line passing through the bottom of sixth cervical vertebra in the neutral/ extension position; *C*_*2*_*C*_*6*_*AR* the ratio between C_2_C_6_Ae and C_2_C_6_An

Binary multivariate logistic regression (backward-Wald) analyses identified two independent correlative factors from the cervical mobility indicators that correlated best as predictors of difficult laryngoscopy: MMT and C_2_C_6_AR. The odds ratio (OR) and 95 % CI were 2.292 (1.093–4.803) and 0.493 (0.306–0.793), respectively (Table 2).

**Table 2:** Cervical mobility predictors for difficult laryngoscopy identified by binary multivariate logistic regression (backward-Wald) model

Variable	Β	SE	*P*-value	OR	95 % CI
MMT	0.829	0.378	0.028	2.292	1.093–4.803
C_2_C_6_AR	-0.708	0.243	0.004	0.493	0.306–0.793
Constant	-1.566	0.661	0.018	0.209	

*SE* standard error; *OR* Odds ratio; *95 %CI* 95 % confidence interval

The AUC and standard error calculated for those clinical tests are shown in Table 3. We used the ROC curve and AUC to identify the predictive abilities of these predictors. C_2_C_6_AR exhibited the largest area under the curve (0.714; 95 % CI 0.633–0.794).

**Table 3:** Predictive values of cervical mobility indicators for predicting difficult laryngoscopy

Indicators	AUC	95 % CI	SE	*P*-value
BMI	0.574	0.487–0.661	0.044	0.136
MMT (class I-II/class III-IV)	0.586	0.489–0.684	0.050	0.081
HMDn (cm)	0.569	0.466–0.671	0.052	0.183
HMDe (cm)	0.586	0.492–0.679	0.048	0.102
HMDR	0.520	0.423–0.617	0.049	0.703
C_0_C_1_Dn (mm)	0.541	0.436–0.647	0.054	0.404
C_0_C_1_De (mm)	0.571	0.482–0.660	0.045	0.152
C_0_C_1_DR	0.524	0.433–0.616	0.047	0.624
C_1_C_2_Dn (mm)	0.555	0.451–0.659	0.053	0.266
C_1_C_2_De (mm)	0.554	0.459–0.649	0.049	0.277
C_1_C_2_DR	0.513	0.415–0.610	0.050	0.796
**C**_**2**_**C**_**6**_**An (°)**	**0.629**	**0.533–0.724**	**0.049**	**0.013**
C_2_C_6_Ae (°)	0.530	0.421–0.638	0.055	0.568
**C**_**2**_**C**_**6**_**AR**	**0.714**	**0.633–0.794**	**0.041**	**< 0.001**

*AUC* area under the curve

A prefer cut-off value should take both sensitivity and specificity into account, therefore, we used the Youden index (sensitivity + specificity-1) to screen out the best cut-off value. When the Youden index took the maximum value of 0.39, the cut-off value of C_2_C_6_AR was set to 1.48, with the sensitivity and specificity was 0.64 and 0.76 respectively. We got rid of the sensitivity values less than 0.60 and specificity values less than 0.3 (Table 4). In clinical application, we pay more attention to screen out the most potential difficult laryngoscopy patients with prefer sensitivity value, the cut-off value of C_2_C_6_AR was set to 1.36, with the sensitivity of 0.71 and the specificity of 0.60.

**Table 4:** Calculated cut-off values that show the best range of sensitivity and specificity for the C_2_C_6_AR

Cut-off point	Sensitivity	Specificity	Youden index
1.48	0.64	0.76	0.40
1.45	0.67	0.70	0.37
1.42	0.68	0.65	0.33
1.36	0.71	0.60	0.31
1.27	0.75	0.54	0.29
1.26	0.76	0.51	0.27
1.21	0.78	0.43	0.21
1.16	0.81	0.41	0.22
1.14	0.82	0.38	0.20
1.04	0.87	0.35	0.22
1.00	0.88	0.30	1.18

Youden index = sensitivity + specificity-1

## Discussion

Most anesthesiologists have experienced difficult airway management, which handled improperly could cause anesthesia-related deaths. There are many physical examinations used to identify people at high risk of difficult airway or difficult intubation, and the upper lip bite test was considered as the most valuable bedside screening test for predicting difficult intubation in general populations [12, 13]. But none of the common bedside tests is well suited for detecting difficult airways for patients with cervical spondylosis, who were reported having a high incidence of difficult laryngoscopy. Although the video laryngoscope provides indirect visualization of the glottis and might be easier to operate, it did not yield a higher first-attempt tracheal intubation success rate than direct laryngoscopy [14, 15]. Therefore, we studied the predictors of cervical mobility indicators which can reflect the difficult laryngoscopy in patients with cervical spondylosis using direct Macintosh laryngoscopy. We compared several cervical mobility indicators including the Body Mass Index (BMI), MMT, the hyomental distance (HMD), and found that C_2_C_6_AR was the best indicator associated with difficult laryngoscopy in patients with cervical spondylosis.

A significantly greater proportion of difficult laryngoscopy and tracheal intubation had been found in obese patients [16, 17]. However, in our study, we found there was no significant difference between the easy and difficult groups (25.1 ± 3.3 vs. 25.7 ± 2.5, *P* = 0.261) which were in accordance with the study reported by Prakash et al [18]. MMT is the most popular test for preoperative airway evaluation which could reflect oropharyngeal cavity volume, but its disadvantage is that it could not adequately reflect laryngeal condition and cervical mobility. In our study, we found MMT had low AUC (0.586) which indicated that MMT might not be a preferred predictor for patients with cervical spondylosis.

HMD measurements in different positions might reflect cervical mobility. Suyama et al [19] presented earlier the test for predicting the difficult intubation airway in 476 patients excluding those with neck disease and anatomical abnormalities and they found that HMD less than 3.0 cm could predict a difficult airway. Based on HMD, HMDR was developed for reflecting neck extension. Takenaka et al. firstly introduced HMDR measured by goniometer in patients with rheumatoid arthritis for evaluation of reduced occipitoatlantoaxial extension capacity [20]. HMD and HMDR can also be measured with the help of ultrasonography. HMD is measured between the anterior border of the chin and the anterior border of the hyoid [21]. In the study by Petrisor et al [22], HMDR seemed to have superior diagnostic accuracy with a cut-off value of 1.23 provides 100 % (39.8–100.0) sensitivity and 90.5 % (69.6–98.8) specificity for the prediction of difficult airway in the obese population.

In our study, we measured HMDn, HMDe, and HMDR by preoperative X-ray, which might be more accurate than ultrasound in the evaluation of skeleton structure. However, we found that they were not significantly different between the easy and difficult laryngoscopy groups, respectively. The median of HMDR in the easy laryngoscopy group was 1.21 which was smaller than the median of HMDR (1.34) in the study by Petrisor et al. However, the median of HMDR in the difficult laryngoscopy group was 1.22 which was in accordance with the median of HMDR (1.21) in the study by Petrisor et al [22]. Our results were different from those of previous studies, which might be related to the following two reasons. Firstly, in our study, all participants were cervical spondylosis patients with abnormal lower cervical spines below hyoid level. Secondly, the HMDR measured by ultrasound in other studies could not eliminate the influence of soft tissue on the indicator measurement. When the boundary of soft tissue and skeleton structure is not clear, the measurement results will have errors.

Patients with atlantooccipital distance impairment had a higher prevalence of difficulty laryngoscopy [23]. Basaranoglu et al [24] conducted a study for 239 patients with an emergency cesarean section, and they found that atlantooccipital extension could not predict difficult tracheal intubation. In our study, there was no significant difference between the easy and difficult laryngoscopy groups in C_0_C_1_Dn, C_0_C_1_De, and C_0_C_1_DR which were consistent with theirs. C_0_C_1_D and C_0_C_1_DR might not be suitable indicators for patients with cervical spondylosis.

Xu et al [25] created a new combined model including radiological indicators to predict the difficult airway. In their study, atlantoaxial distance had no significant difference between the easy and difficult laryngoscopy groups (4.6 ± 1.0 vs. 4.7 ± 1.1, *P* = 0.542). In our study, the result was in line with Xu et al. and we found that C_1_C_2_Dn, C_1_C_2_De and C_1_C_2_DR were not significantly different between the easy and difficult laryngoscopy groups, respectively: C_1_C_2_Dn [4.6(2.5)mm vs. 5.0(4.3)mm; *P* = 0.266], C_1_C_2_De [0.5(0.3)mm vs. 0.6(0.3)mm; *P* = 0.277], C_1_C_2_DR [8.88(5.36) vs. 8.96(6.51); *P* = 0.796]. It needs further researches to find out suitable distance index reflecting the activity of cervical spine mobility for predicting difficult laryngoscopy.

The angle from C2-C6 seen in our study implied the limited flexion of lower cervical spines, which might result in difficult laryngoscopy. Under such circumstances, indicators reflecting lower cervical spine mobility may have a better prediction. In our study, we found that C_2_C_6_Ae was not a valuable indicator for predicting difficult laryngoscopy in patients with cervical spondylosis. However, C_2_C_6_An and C_2_C_6_AR were both effective indicators. C_2_C_6_AR was a new predictor and the only independent correlative factor from the cervical mobility indicators for difficult laryngoscopy in cervical spondylosis patients with an AUC of 0.714. More studies are needed to explore and evaluate the application of C_2_C_6_AR as a difficult laryngoscopy predictor to other types of patients.

## Limitations

Our study had some limitations. The best cut-off-point of C_2_C_6_AR, as a predictor of difficult laryngoscopy, was determined and analyzed both in the same population. We didn’t recruit another group of patients for external validation. Besides, the results of our study applied to patients just with cervical spondylosis, and the extension of the present results warrants further investigation.

## Conclusions

C_2_C_6_AR based on preoperative X-ray images could be a valuable radiologic predictor of cervical mobility indicators for difficult laryngoscopy in patients with cervical spondylosis.

## Data Availability

The data used to support the findings of this study are available upon reasonable request via e-mail with the corresponding authors.

## Abbreviations

MMT: Modified Mallampati test
BMI: Body Mass Index
HMD: The hyomental distance
HMDn/e: The distance between the hyoid bone and the tip of the chin in the neutral/extension position
HMDR: The ratio between HMDe and HMDn
C_0_C_1_Dn/e: The distance between the occipital bone and the first cervical vertebra in the neutral/extension position
C_0_C_1_DR: The ratio between C_0_C_1_Dn and C_0_C_1_De
C_1_C_2_Dn/e: The distance between the first cervical vertebra and the second cervical vertebra in the neutral/extension position
C_1_C_2_DR: The ratio between C_1_C_2_Dn and C_1_C_2_De
C_2_C_6_An/e: The angle between a line passing through the bottom of second cervical vertebra and a line passing through the bottom of sixth cervical vertebra in the neutral/extension position
C_2_C_6_AR: The ratio between C_2_C_6_Ae and C_2_C_6_An
SD: Standard deviation
SE: Standard error
IOR: The median and interquartile range
OR: Odds ratio
CI: Confidence interval
ROC: Receiver operating characteristic
AUC: Area under the curve
